# Comparison of Sharp Dissection, Electrocautery, and Ultrasonic Activated Scalpel with Regard to Endothelial Damage, Preparation Time, and Postoperative Bleeding During Radial Artery Harvesting

**DOI:** 10.21470/1678-9741-2018-0311

**Published:** 2019

**Authors:** Dinçer Uysal, Şenol Gülmen, Hayrettin Özkan, Ulaş Sağlam, Mustafa Etli, Sema Bircan, Recep Sütçü, Turhan Yavuz, Hakan Öntaş, Fatih Aksoy

**Affiliations:** 1 Department of Cardiovascular Surgery, Suleyman Demirel University, School of Medicine, Isparta, Turkey.; 2 Department of Pathology, Suleyman Demirel University, School of Medicine, Isparta, Turkey.; 3 Department of Biochemistry, Suleyman Demirel University, School of Medicine, Isparta, Turkey.; 4 Department of Cardiology, Suleyman Demirel University, School of Medicine, Isparta, Turkey.

**Keywords:** Radial Artery, Vascular Cell Adhesion Molecular, Nitric Oxide, Coronary Artery Bypass, Electrocoagulation, Drainage, Staining and Labeling

## Abstract

**Objective:**

To examine the effects of classical technique, electrocautery, and ultrasonic dissection on endothelial integrity, function, and preparation time for harvesting the radial artery (RA) during coronary artery bypass grafting (CABG).

**Methods:**

Forty-five patients who underwent isolated CABG and whose RA was suitable for use were studied and divided into three groups: Group 1, classical method (using sharp dissection); Group 2, electrocautery; and Group 3, ultrasonic cautery. Levels of prostacyclin and nitric oxide derivatives were examined biochemically; vascular cell adhesion molecule 1 (VCAM-1) and endothelial nitric oxide synthetase (eNOS) values were assessed using immunohistochemical staining. RA preparation time, RA length/harvesting time ratio, and drainage amounts at the site of RA removal were compared.

**Results:**

Differences in RA preparation time (Group 1: 25±6 min, Group 2: 18±3 min, Group 3: 16±3 min, *P*<0.001) and length/harvesting time ratio (Group 1: 0.76±0.19 cm/min, Group 2: 0.98±0.16 cm/min, Group 3: 1.13±0.09 cm/min, *P*<0.001) were statistically significant among the groups. Levels of prostacyclin and nitric oxide derivatives were not statistically significant different, VCAM-1 and eNOS expressions were observed to be similar among the groups, and endothelial damage was detected in only one patient per group.

**Conclusion:**

Use of ultrasonic cautery during RA preparation considerably reduces the preparation time and postoperative drainage amount. However, the superiority of one method over the others could not be demonstrated when the presence of endothelial damage with both biochemical and histopathological evaluations was considered.

**Table:** 

Abbreviations, acronyms & symbols	
CABG	= Coronary artery bypass grafting
ELISA	= Enzyme-linked immunosorbent assay
eNOS	= Endothelial nitric oxide synthetase
ICAM-1	= Intracellular adhesion strength molecule 1
IHC	= Immunohistochemical
RA	= Radial artery
SPSS	= Statistical Package for the Social Sciences
VCAM-1	= Vascular cell adhesion molecule 1

## INTRODUCTION

The radial artery (RA) is frequently used in coronary artery surgery for revascularization^[1]^. The advantage of RA as a graft is its adaptation to systemic blood pressure and its large diameter compared to other arterial grafts^[2]^. However, after trauma, RA has a stronger spasmodic response than the mammary artery. Thus, the biggest barrier to the usage of RA is this vasospastic feature that causes a marked reduction in the artery diameter and considerably reduces the blood flow. RA has the characteristics of a type III muscular artery. Disruption of endothelial integrity during RA preparation may prevent endothelium-dependent laxation and may lead to early graft failure^[3-5]^.

Therefore, protection of endothelial features of the RA may be of importance when considering the techniques used during graft preparation. It is often recommended to prepare the RA with the accompanying veins within the pedicle in order to prevent the occurrence of spasm. Harmonic scalpel (Harmonic scalpel^®^, HS, Ethicon Endo-Surgery, Cincinnati, Ohio) and electrocautery (Petaş^®^Profesyonel Elektronik, Ankara, Turkey) are reported to be suitable devices for this technique^[6]^. Skeletonized harvesting of RA using sharp dissection is a less frequently used method^[7,8]^.

This study aimed to compare the effects of the classical technique, electrocautery, and ultrasonic dissection on the endothelial integrity and characteristics of the RA during coronary artery bypass grafting (CABG).

## METHODS

This study was approved by the local institutional review board (ethical committee date: 06.9.2007; session no: 07; decision number: 03), and written consent was obtained from each patient before the study.

The study sample consisted of 45 candidates for CABG with good flow to the palmar arch of the nondominant hand. The patients were randomly divided into three groups and matched for demographic characteristics. The RA was harvested with hemostatic clips, scissors, and minimal electrocautery in Group 1 (n=15), with electrocautery in Group 2 (n=15), and with the Harmonic scalpel in Group 3 (n=15).

Allen’s test with pulse oximetry was used to assess the adequacy of blood supply from the ulnar artery to the nondominant hand. None of the patients had any contraindication for RA harvest, defined as a delay in capillary refill exceeding 10 seconds. If the test results were negative, a modified Allen’s test was carried out to confirm the result. During the same procedure, oxygen saturation of the thumb was measured by means of a pulse oximeter. The patients that showed inadequate blood supply by either Allen’s or the modified Allen's test were excluded from the study. Additionally, the exclusion criteria included patients with concomitant valve surgery, trauma in the arm from which the RA would be harvested, arteriovenous fistula, chronic kidney failure, raynaud’s disease, collagen tissue disease, anatomic vascular anomaly in the upper extremity, bleeding diathesis, and those who refused the procedure.

### Surgical Procedure

All patients underwent infusion with diltiazem (Diltizem^®^ 25 mg, Mustafa Nevzat) in order to prevent spasm of the RA after anesthesia induction. The same surgeon (D.U.) carried out the removal the RA vessels in all patients for standardization of the technique. An incision was made from the wrist (over the RA pulse) to the mid-antecubital fossa (over the brachial artery pulse). A surgical blade was used only for the skin incision. In the Group 1, scissors were used to separate the RA from the subcutaneous tissue, muscle, and overlying fascia. Low-voltage electrocautery was used for hemostasis in the subcutaneous tissue, but bleeding control was ensured by tying with 4/0 silk in the deep tissue. Collateral branches of the RA were tied with double clips or 4/0 silk and the middle parts were cut with scissors. No electrocautery or ultrasonic cautery was used adjacent to the RA. The two satellite veins and the surrounding adipose tissues were not removed during the procedure. In the Group 2, low-energy electrocautery (Petaş^®^Profesyonel Elektronik, Ankara, Turkey) was used to separate the RA from the subcutaneous tissue, muscle, and overlying fascia. The collateral branches were occluded with hemostatic clips (Vitalitec^®^, Domalain, France) and divided with scissors. Electrocautery was avoided to prevent thermal injury to the artery and to ensure patency of the arterial grafts. In addition, no metallic probes or dilators were used in order to prevent intimal trauma. The two satellite veins and the surrounding adipose tissues were not removed from the patients in this group either. In the Group 3, an ultrasonic cautery (Harmonic Scalpel^®^, HS, Ethicon Endo-Surgery, Cincinnati, Ohio) with coagulating curved shears and 14 cm scissor-grip handle was used to cut the subcutaneous tissue and overlying fascia of the RA. After the antebrachialis fascia was incised, a self-retaining retractor was placed between the brachioradialis and flexor carpi radialis muscles in order to expose the entire length of the RA. The two satellite veins and the surrounding adipose tissue were left attached to the RA to preserve its blood supply as much as possible and to prevent spasm. The RA and the side branches of the satellite vein were coagulated with the coagulating shears of the ultrasonic scalpel along their entire length. The shears were then used to free the medial side from the adjacent tissue and to apply minimal upward traction to the underside of the RA. Finally, the lateral side was dissected, and the RA pedicle was ligated and divided at both ends. In all groups, the RA graft was stored in a solution containing blood, normal saline, and heparin. Hemostasis was carefully verified before the arm incision was closed. The fascia antebrachialis was left open to prevent compartment syndrome. The drainage time was initiated after closing the surgical incision line. The amount of drainage was measured after 24 and 48 hours. Dressings and elastic bandages were applied, and the arm was repositioned parallel to the patient’s body by using an elbow pad. The arm and hand were re-examined before the patient was transferred from the operating room.

The groups were compared for harvesting time (interval from skin incision to ligation of the proximal end of the artery), total drainage from the incised arm (between closure of the incision and postoperative two days), hematomas (requiring re-exploration of the arm), superficial wound infection (managed with local drainage and antibiotics), and endothelial function.

One half of the 1 cm tissue taken from the RA end was placed in an Eppendorf tube with phosphate buffer. The tissue was delivered to the biochemistry laboratory within 24 hours for biochemical examination of endothelium damage and stored in the lab at -20º C until use. The other half of the tissue was placed in a capped Eppendorf tube containing 10% formaldehyde solution for histopathological examination of endothelium damage. After the tissues were collected, all samples were delivered to the pathology laboratory. To measure the drainage, a minivac with an output hole was placed about 1 cm lateral to the most distant end of the RA incision. Drainage amount was recorded until the postoperative day two, following the closure of the skin.

### Biochemical Examination

The samples were defrosted at room temperature, individually weighed, and spiked 1/9 with phosphate buffer, pH 7.4. The tissues were crushed with a macrotome first, and then with microtomes. Following this, the samples were homogenized with a homogenizer at 10,000 cycle/min on ice, sonicated for 30 seconds, followed by centrifugation. The resulting supernatant was measured for prostacyclin levels by enzyme-linked immunosorbent assay (ELISA) (monoclonal human specific ELISA kit, Oxford^®^, USA) and for nitric oxide levels by a spectrophotometric method (human specific kit, Biovision^®^, USA).

### Histopathological Examination

Tissue samples taken from the distal end of the harvested RA were fixed for 24 hours in a 10% buffered neutral formaldehyde solution. All samples were taken for routine tracking on a tissue tracking device (Shandon Pathcenter^®^) and paraffin blocks were prepared. Serial sections of 5 µm thickness were taken for each tissue sample from these paraffin blocks using a microtome (Leica Rotary^®^) and were stained for immunohistochemistry. Histopathological examination was performed with a light microscope.

### Immunohistochemical (IHC) Examination

The sections were prepared at 0.4 micron thickness and blocked with Ultra V block (LabVision, TA-125-UB^®^). Deparaffinized sections were evaluated by IHC for the expression of intracellular adhesion strength molecule 1 (ICAM-1) and endothelial nitric oxide synthetase (eNOS). For this, the sections were incubated with 1/100 dilution of an ICAM-1 antibody (sc-107, Santa Cruz Biotechnology, CA^®^, USA) for 60 min and 1/50 dilution of an eNOS antibody (sc-654, Santa Cruz Biotechnology, CA^®^, USA) for 60 min.

The stain retention and intensity for the IHC experiments were graded as -, +, and ++ (-, no staining; +, slight staining; and ++, severe staining), with reference to the scoring system by Shapira et al.^[9]^ IHC findings were graded as follows: a score of 0 was assigned to (-) indicating no endothelial staining; score of 1 was assigned to (+) indicating faint staining across the entire endothelium, staining was seen at 10x zoom under a light microscope; while a score of 2 was assigned to (++) indicating marked staining across the entire endothelium, staining was seen even at 4x zoom under a light microscope. All pathological examinations were carried out by a pathologist blinded to the study design.

### Statistical Analysis

Statistical analysis was performed using the Statistical Package for the Social Sciences (SPSS) software, version 15.0 (SPPS Inc., California, IL, USA). Data were expressed as mean ± standard deviation. Kruskal-Wallis test was used to determine the differences between all groups and Mann-Whitney U test was used to determine the difference between two groups. P≤0.05 was considered as statistically significant.

## RESULTS

The patients’ mean age was 59.8±8.2 years (range: 45-77 years), and there was no statistically significant difference between the groups. Thirty-four patients were males (75.6%) and 11 were females (24.4%). The patient’s demographic and clinical variables are listed in Table 1. None of the patients underwent revision surgery due to bleeding from the forearm. No signs of ischemia were detected intraoperatively and postoperatively in the arm where the RA was harvested.

**Table 1:** Patients’ demographic and clinical characteristics.

Variables		Group 1 (n=15)	Group 2 (n=15)	Group 3 (n=15)	*P*-value
Sex, males/females		11-4	12-3	11-4	0.886
Age, years		61.7±7 (range, 45-71)	57.3±9.6 (range, 45-77)	60 & #x00b1; 6.6 (range, 53-74)	0.519
Comorbiditiy, N (%)	Hypertension	9 (60.0)	7 (46.7)	11 (73.3)	0.329
	Diabetes mellitus	11 (73.3)	10 (66.7)	9 (60.0)	0.748
	Dyslipidemia	10 (66.7)	8 (53.3)	12 (80.0)	0.301
	Alcohol	1 (6.7)	2 (13.3)	1 (6.7)	0.760
	Smoker	8 (53.3)	7 (46.7)	6 (40.0)	0.464
Coronary artery disease					0.649
	2-vessel disease	3	2	4	
	3-vessel disease	12	13	11	

### Biochemical Findings

The amount of prostacyclin derivatives was found to be 42.97±29.7 ng/g protein (6.74-98.65) in Group 1, 60.1±25.6 ng/g protein (15.6-111.24) in Group 2, and 54.8±31.07 ng/g protein (6.24-98.65) in Group 3 (*P*=0.45). Nitric oxide derivatives were detected as 17±17.1 µmol/g protein (1.4-49.4) in Group 1, 5.2±2.5 µmol/g protein (1.9-10.6) in Group 2, and 11.2±9.7 µmol/g protein (2.4-31.9) in Group 3 (*P*=0.17). There were no significant differences in the levels of these derivatives between the groups in the context of endothelial damage.

### Histopathological Findings

**Fig. 1:**
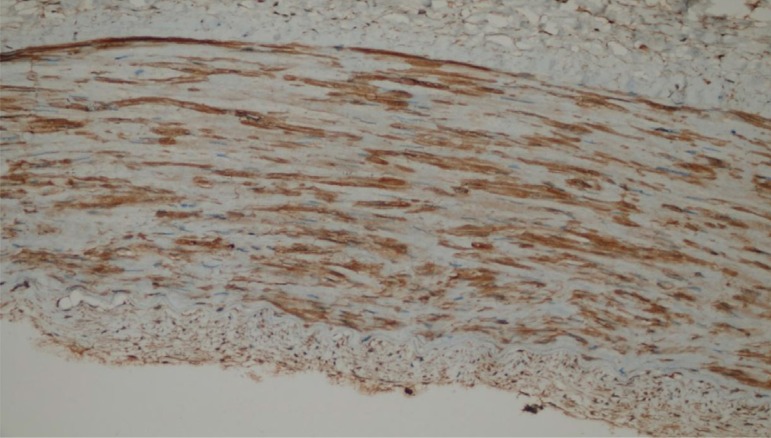
Endothelial nitric oxide synthetase (eNOS) of patient no. 17 (in the electrocautery group) ++ endothelial damage (eNOS immunohistochemical staining of the transverse section taken from the radial artery sample) (eNOS immunoreactivity in endothelial cells ++) (40×).

**Fig. 2:**
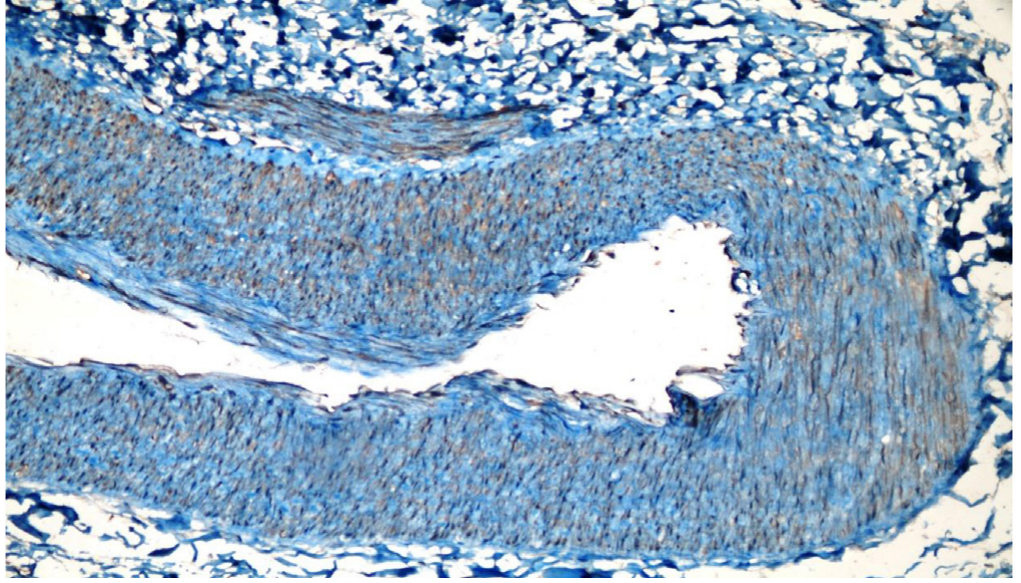
Endothelial nitric oxide synthetase (eNOS) of patient no. 22 (in the sharp dissection group) + endothelial damage (eNOS immunohistochemical staining of the transverse section taken from the radial artery sample) (eNOS immunoreactivity in endothelial cells +) (10×).

**Fig. 3:**
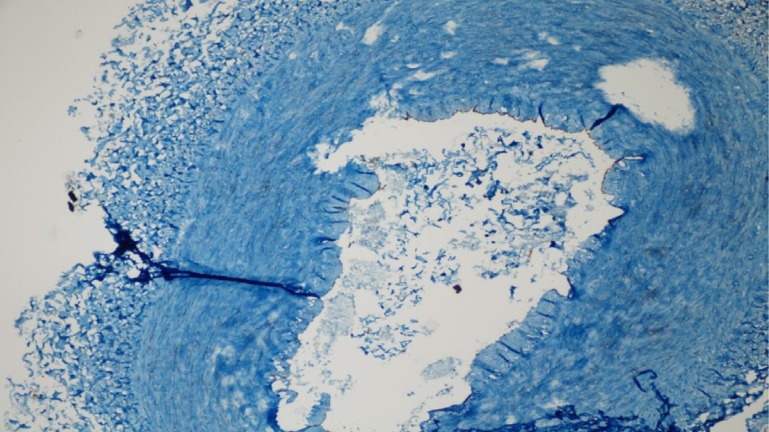
Intracellular adhesion strength molecule (ICAM) of patient no. 30 (in the ultrasonic cautery group) – endothelial damage (ICAM immunohistochemical staining of the transverse section taken from the radial artery sample) (ICAM immunoreactivity in endothelial cells -) (4×).

We next evaluated the endothelial damage by determining the expression of ICAM-I and eNOS by IHC. We observed that only one patient per group showed signs of endothelial damage and there was no significant difference between the groups for endothelial damage (Table 2). The microscopic views of endothelial damage are shown in Figures 1, 2, and 3.

**Table 2:** Statistical analysis of immunohistochemical scores and values between the groups.

Immunohistochemical technique	Group 1				Group 2				Group 3				*P*-value
	-	+	++	Mean score	-	+	++	Mean score	-	+	++	Mean score	*P*
ICAM-1 (n)	10	4	1	-	11	3	1	-	9	5	1	-	0.556
eNOS (n)	11	3	1	-	10	4	1	-	9	5	1	-	0.977

Immunohistochemical findings were graded as follows: A score of 0 was assigned to (-) indicating no endothelial staining; a score of 1 was assigned to (+) indicating faint staining across the entire endothelium, staining was seen at 10´ zoom under a light microscope; and a score of 2 was assigned to (++) indicating marked staining across the entire endothelium, staining was seen even at 4´ zoom under a light microscope.

ICAM-1=intracellular adhesion strength molecule 1; eNOS=endothelial nitric oxide synthetase

### Radial Artery Preparation Process and Follow-Up

The average preparation time of RA was 25.1±5.9 min (14.5-33.5) in Group 1, 17.8±2.8 min (11.5- 21.5) in Group 2, and 16±2.6 min (13-21.4) in Group 3. The average length of the RA was 17.5±1.6 cm (15-22) across all the groups. Kruskal-Wallis test indicated that the average preparation times were significantly different between the groups; therefore, a post-hoc analysis was carried out. The preparation time for RA was found to be significantly shorter in Group 2 than in Group 1 (*P*=0.001) and significantly shorter in Group 3 than in Group 1 (*P*=0.001). However, there were no statistical differences in the preparation time between Groups 2 and 3.

**Fig. 4:**
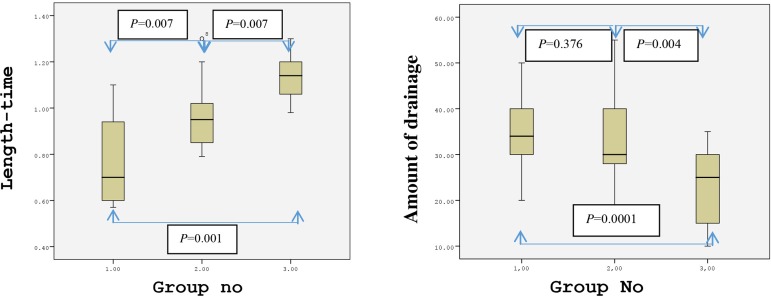
Distribution of length-time ratio between the groups. Group 1=classical method (using sharp dissection); Group 2=electrocautery; Group 3=ultrasonic cautery

We next compared the ratio of RA length/RA preparation time between the groups. This ratio was found to be 0.76±0.19 cm/min (0.5-1.1) in Group 1, 0.98±0.16 cm/min (0.7-1.3) in Group 2, and 1.13±0.09 cm/min in Group 3. Post-hoc tests revealed that the RA length/RA preparation time ratio was significantly higher in Group 2 than in Group 1 (*P*=0.007), in Group 3 than in Group 1 (*P*=0.001), and in Group 3 than in Group 2 (*P*=0.007). The distribution of the length-time ratios between the groups is shown in Figure 4.

**Fig. 5:**
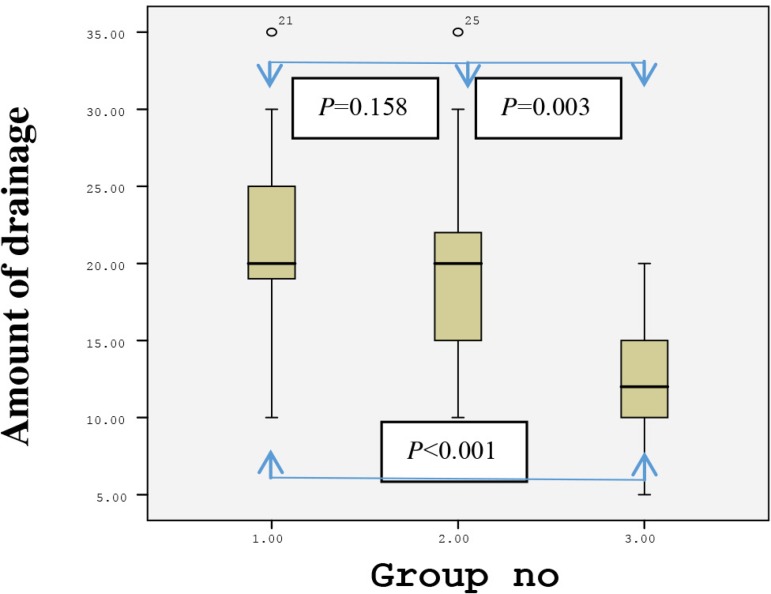
Distribution of 24-hour drainage amount between the groups. Group 1=classical method (using sharp dissection); Group 2=electrocautery; Group 3= ultrasonic cautery

**Fig. 6:**
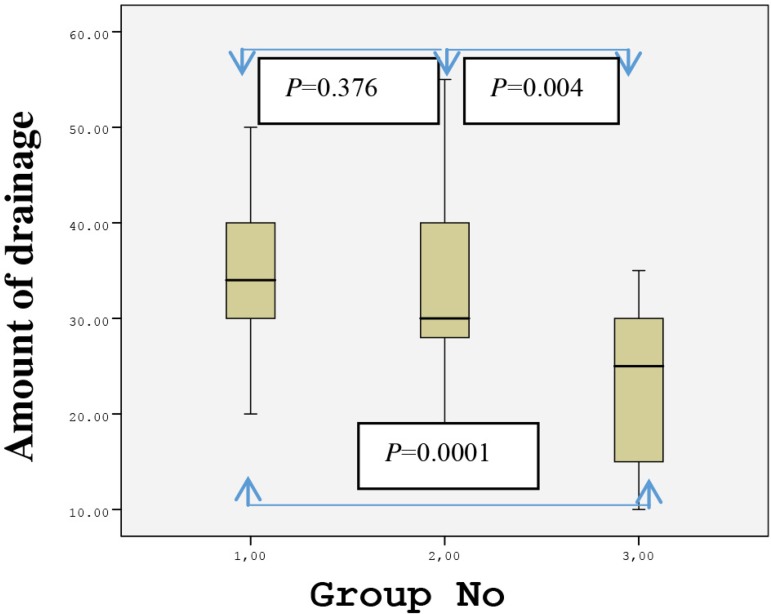
Distribution of 48-hour drainage amount between the groups. Group 1=classical method (using sharp dissection); Group 2=electrocautery; Group 3=ultrasonic cautery

The postoperative forearm drainage amount in the first 48 hours was 35±8.3 ml (20-50) in Group 1, 32.2±10.9 ml (15-55) in Group 2, and 22.3±8.5 ml (10-35) in Group 3. The drainage amount was significantly lower in Group 3 than in Groups 1 and 2 (*P*=0.0001 and *P*=0.004, respectively). The distributions of the postoperative drainage amount between the groups at 24 and 48 hours are shown in Figures 5 and 6. None of the patients underwent any revision surgery due to forearm hemorrhage. Additionally, there were no sign of ischemia during or after the operation in the arm in which the RA excision was carried out.

## DISCUSSION

Although there have been several studies on the use of RA as a graft in CABG in the past 20 years, there are still some unanswered questions on the preparation methods. Thermal injury and mechanical trauma that may occur during graft preparation can affect the early or late opening of the graft while using the RA, which is already prone to spasm, and may cause spasm in the graft, thereby affecting blood flow^[10,11]^.

Protein coagulation with ultrasonic cautery has been commonly used in the recent years. The ultrasonic cautery cuts and coagulates the vascular collateral branches by cavity fragmentation and mechanical incision per vibration, and the vascular collateral functions by dissecting perivascular tissues. The use of this device in the preparation of RA is considered to be less traumatic compared to conventional techniques^[1,12-14]^.

Electrocautery generates more heat compared to ultrasonic cautery, but if it is used minimally and at low voltage at a position close to the RA during dissection, its negative effects on the RA can be prevented^[15]^.

RA preparation with Harmonic scalpel may take less time compared to conventional methods. Georghiou et al.^[16]^ compared the preparation of 100 RAs by randomization using Harmonic and electrocautery methods and detected that the average RA preparation time was 37 min for electrocautery and 20 min for Harmonic scalpel. Wright et al.^[15]^ reported that harvesting of RA using Harmonic scalpel required a significantly lower number of clips to control the bleeding than using electrocautery. There was no significant difference between the times required to harvest the artery with either device. Moreover, there were no complications, malfunctions, or serious adverse events associated with the use of either device.

Posacioglu et al.^[17]^ reported the RA preparation time of 20 min with Harmonic scalpel and 36 min with electrocautery in 20 patients. In another study with 40 patients, RA preparation time was 17.6±4.8 min in the ultrasonic cautery group and 21.8±3.2 min in the electrocautery group (*P*<0.05). The calculated RA length/preparation time ratio was 1±0.11 cm/min and 0.84±0.06 cm/min, respectively (*P*<0.05). Drainage amount from the forearm was measured to be 31.5±9.4 mL (15-45) and 34.5±11 mL (15-55) for the two methods, respectively.

Canosa et al.^[18]^ harvested the RA with ultrasonic cautery in 20 patients and found the RA preparation time to be 10.3 min (7-15). Chukwuemeka et al.^[19]^ compared sharp dissection with electrocautery and reported that the preparation time was 34.4±4.7 min in the sharp dissection group and 18.8±2.4 min in the electrocautery group.

In a study with 20 patients, Tarhan et al.^[6]^ compared the use of scissors, electrocautery, and ultrasonic cautery for harvesting time, spasm, and electron microscopic endothelium examination and found all the results between the groups to be similar.

Erkut et al.^[20]^ reported a comparison of preparation time, hemostatic clip usage, spasm, in situ flow, and endothelial damage in 101 patients undergoing grafting. Endothelial damage in the RA taken with Harmonic scalpel was significantly smaller than with high-frequency electrocautery (*P*<0.05). There was no statistically significant difference in the harvesting time and spasm between the two groups (*P*>0.05). As a result, it was concluded that ultrasonic dissection with Harmonic scalpel of the RA was associated with decreased use of hemostatic clips. These authors reported that the Harmonic scalpel had a positive effect on endothelial preservation and was associated with increased free blood flow of the RA.

Marzban et al.^[3]^ compared electrocautery and sharp dissection in a study involving 44 patients and found no differences between the two groups in terms of endothelial damage; the preparation time was found to be shorter in the electrocautery group than in the sharp dissection group. In the present study, patients in Group 3 showed a significantly higher rate of endothelial damage than those in Groups 1 and 2. Although there was no significant difference in the preparation time of RA between Group 2 and Group 3, we attributed the statistically significant difference in the length/time ratio in Group 3 to the presence of longer RAs in the patients of this group than those of Groups 1 and 2.

Rukosujew et al.^[21]^ divided 40 patients into four groups: Group 1 for skeletonized sharp dissection, Group 2 for pedicled sharp dissection, Group 3 for skeletonized ultrasonic cautery, and Group 4 for pedicled ultrasonic cautery. The graft preparation times were reported to be 37.1±3.5 min in Group 1; 24.4±3.9 min (*P*<0.001) in Group 2; 31.1±3.5 min in Group 3; and 25.6±3.7 min in Group 4 (*P*<0.01). The differences in preparation times may be due to the visualization of all collateral branches when preparing the skeletonized grafts and working near the RA. In the current study, the RA preparation was significantly shorter with ultrasonic cautery than with other methods, which is a finding consistent with the literature^[3,15,16]^.

Increased prostacyclin and nitric oxide levels in the endothelial tissue are indicative of endothelial damage^[22,23]^. Shapira et al.^[9]^ compared ultrasonic cautery, electrocautery, and the endoscopic method in a study with 54 patients and found the expression of adhesion molecule (ICAM-1, vascular cell adhesion molecule 1 [VCAM-1], P-Selectin) as well as histological findings under both light and electron microscope to be similar. The findings of the current study corroborate these data and indicate that there was no statistically difference in the levels of prostacyclin and nitric oxide derivatives among the different groups. Again, when endothelial damage was evaluated by the expression of ICAM-1 and eNOS by IHC, we did not observe any statistical difference between the groups. This may be due to the meticulous care taken during the preparation of the RA in all three groups.

## CONCLUSION

In conclusion, in the current study, we showed that the RA was harvested significantly faster using ultrasonic cautery than using sharp dissection and electrocautery, and its postoperative drainage amount was smaller than theirs. Additionally, none of the methods was superior to the other in the context of endothelial damage when examined with both histopathological and biochemical techniques.

**Table:** 

Authors’ roles & responsibilities	
DU	Substantial contributions to the conception or design of the work; or the acquisition, analysis, or interpretation of data for the work; final approval of the version to be published
SG	Article review; final approval of the version to be published
HO	Article review; final approval of the version to be published
US	Article review; final approval of the version to be published
ME	Article review; final approval of the version to be published
SB	Pathological analysis; final approval of the version to be published
RS	Biochemical analysis; final approval of the version to be published
TY	Conception and design of the project; data collection; discussion of results; article review; final approval of the version to be published
HO	Article review; final approval of the version to be published
FA	Conception and design of the project; data collection; discussion of results; article review; final approval of the version to be published

## References

[1] Risteski PS, Akbulut B, Moritz A, Aybek T (2006). The radial artery as a conduit for coronary artery bypass grafting: review of current knowledge. Anadolu Kardiyol Derg.

[2] Cikirikcioglu M, Posacioglu H (2006). Harmonic scalpel has acute beneficial effects during radial artery harvesting. Asian Cardiovasc Thorac Ann.

[3] Marzban M, Arya R, Mandegar MH, Karimi AA, Abbasi K, Movahed N (2006). Sharp dissection versus electrocautery for radial artery harvesting. Texas Hear Inst J.

[4] Dogan ÖF, Khalil E, Kara KA, Duman Ü (2018). ?Aminoacid enriched cardioplegia decreased the amount of intramyocardial leucocytes in coronary artery bypass grafting surgery. Am J Cardiol.?.

[5] Brazio PS, Laird PC, Xu C, Gu J, Burris NS, Brown EN (2008). Harmonic scalpel versus electrocautery for harvest of radial artery conduits: reduced risk of spasm and intimal injury on optical coherence tomography. J Thorac Cardiovasc Surg.

[6] Tarhan IA, Kehlibar T, Arslan Y, Yilmaz M, Dumantepe M, Berkoz K (2007). Effects of normothermic organ bath and verapamil-nitroglycerin solution alone or in combination on the blood flow of radial artery. Eur J Cardiothorac Surg.

[7] Rukosujew A, Reichelt R, Fabricius AM, Drees G, Tjan TD, Rothenburger M (2004). Skeletonization versus pedicle preparation of the radial artery with and without the ultrasonic scalpel. Ann Thorac Surg.

[8] Fawzy HF (2009). Harvesting of the radial artery for coronary artery bypass grafting: comparison of ultrasonic harmonic scalpel dissector with the conventional technique. J Card Surg.

[9] Shapira OM, Eskenazi BR, Anter E, Joseph L, Christensen TG, Hunter CT (2006). Endoscopic versus conventional radial artery harvest for coronary artery bypass grafting: functional and histologic assessment of the conduit. J Thorac Cardiovasc Surg.

[10] Royse AG, Royse CF, Tatoulis J, Grigg LE, Shah P, Hunt D (2000). Postoperative radial artery angiography for coronary artery bypass surgery. Eur J Cardiothorac Surg.

[11] Emir M, Gol MK, Ozisik K, Bakuy V, Sargon MF, Yavas S (2004). Harvesting techniques affect the integrity of the radial artery: an electron microscopic evaluation. Ann Thorac Surg.

[12] Ronan JW, Perry LA, Barner HB, Sundt 3rd TM (2000). Radial artery harvest: comparison of ultrasonic dissection with standard technique. Ann Thorac Surg.

[13] Budillon AM, Nicolini F, Agostinelli A, Beghi C, Pavesi G, Fragnito C (2003). Complications after radial artery harvesting for coronary artery bypass grafting: our experience. Surgery.

[14] Patel A, Asopa S, Dunning J (2006). Does radial artery harvest with a harmonic scalpel result in fewer complications than standard electrocautery methods. Interact Cardiovasc Thorac Surg.

[15] Wright CB, Barner HB, Gao A, Obial R, Bandy B, Perry L (2001). The advantages of the harmonic scalpel for the harvesting of radial arteries for coronary artery bypass. Heart Surg Forum.

[16] Georghiou GP, Stamler A, Berman M, Sharoni E, Vidne BA, Sahar G (2005). Advantages of the ultrasonic harmonic scalpel for radial artery harvesting. Asian Cardiovasc Thorac Ann.

[17] Psacioglu H, Atay Y, Cetindag B, Saribülbül O, Buket S, Hamulu A (1998). Easy harvesting of radial artery with ultrasonically activated scalpel. Ann Thorac Surg.

[18] Canosa C, Nasso G, De Filippo CM, Modugno P, Spatuzza P, Calvo E (2007). Open clip-free radial artery harvesting with the harmonic shears. J Card Surg.

[19] Chukwuemeka AO, Deshpande R, Desai JB (2003). Modified technique for rapid atraumatic radial artery harvesting. J Card Surg.

[20] Erkut B, Unlu Y, Karapolat S, Ugur Kocogullari C, Ceviz M, Becit N (2008). Comparison of harmonic scalpel and high-frequency electrocautery in radial artery harvesting. J Cardiovasc Surg (Torino).

[21] Rukosujew A, Hoffmeier A, Rothenburger M, Löher A, Etz C, Ghezelbash F (2005). Harvest of the radial artery: technique of the skeletonization and pedicle preparation. J Cardiovasc Surg (Torino).

[22] Sayers RD, Watt PA, Muller S, Bell PR, Thurston H (1992). Endothelial cell injury secondary to surgical preparation of reversed and in situ saphenous vein bypass grafts. Eur J Vasc Surg.

[23] Shen LZ, Chen XJ, Chen X, Xu M, Wang LM, Jiang YS (2010). The morphometry and eNOS expression of radial artery in elderly patients with coronary atherosclerotic heart disease. Zhonghua Wai Ke Za Zhi.

